# Common coupled fixed point theorems satisfying rational type contractive conditions in b-metric spaces

**DOI:** 10.1186/s40064-016-1849-6

**Published:** 2016-03-02

**Authors:** Muhammad Sarwar, Saddam Hussain, Panda Sumati Kumari

**Affiliations:** Department of Mathematics, University of Malakand, Chakdara Dir(L), Pakistan; Department of Mathematics, National Institute of Technology-Andhra Pradesh, Tadepalligudem, India

**Keywords:** Complete b-metric space, Common fixed point, Coupled fixed point, Coupled coincidence point, Rational type contractive conditions, Primary 47H10; Secondary 54H25

## Abstract

In this article, existence and uniqueness of common coupled fixed point for a pair of mappings in the setup of complete b-metric spaces are studied. The derived result generalizes and extends some well known results from the existing literature in b-metric spaces. Appropriate example is also given.

## Background

Bakhtin (1989) and Czerwik (1993) generalized the notion of metric spaces and introduced the concept of b-metric spaces, which is also known as metric type space (Hussain et al. 2012). b-metric space solved some problems, particulary the problem of the convergence of measurable functions with respect to a measure, lead to a generalization of notation of metric. Using this concept Czerwik (1993, 1998), generalized the well known Banach contraction principle in b-metric spaces, see Czerwik (1998), Czerwik et al. (1997, 2001). Many researchers including Aydi et al. (2012), Boriceanu (2009a, b, c), Bota et al. (2011), Chugh et al. (2012), Shih Du and Karapnar (2013), Kir and Kiziltunc (2013), Olaru and Branga (2011), Olatinwo and Imoru (2008), Lina and Curar (2010) and Pacurar (2010) studied the extension of fixed point theorems in b-metric space.

Guo and lakshmikantham (1987) introduced the concept of coupled fixed point for partially ordered set. By using the concept of mixed monotone property (Gnana Bhaskar and Lakshmikantham 2006) studied the existence and uniqueness of a coupled fixed point result in partially ordered metirc space. After that many researchers studied the coupled fixed point and discussed it’s application. See Berinde (2012), Gnana Bhaskar and Lakshmikantham (2006), Guo and lakshmikantham (1987), Mustafa et al. (2013), Mustafa et al. (2014), Mustafa et al. (2014), Sintunavarat et al. (2012), Sintunavarat et al. (2013). Recently Malhotra and Bansal (2015) studied the existence and uniqueness of common coupled fixed points for a pair of mappings in complete b-metric space.

The aim of this manuscript is to study the existence and uniqueness of common coupled fixed point for a pair of mappings in the setup of complete b-metric space. The derived results generalizes some well known results from the existing literature.

## Preliminaries

Throughout this paper ${{\mathbb {R}}}$ is the set of real and ${{\mathbb {R}}}^{+}$ is set of positive real numbers.

### **Definition 1**

(Bakhtin 1989; Boriceanu 2009c) Suppose *X* be a non empty set and $s\ge 1,s\in {{\mathbb {R}}}$. A function $d:X\times X\rightarrow {{\mathbb {R}}}^{+}$ is said to be b-metric if for all $x,y,z\in X$, the following condition are satisfied:

1. $$d(x,y)=0\Leftrightarrow x=y;$$
2. $$d(x,y)=d(y,x);$$
3. $$d(x,z)\le s[d(x,y)+d(y,z)].$$

Then the pair (*X*, *d*) with parameter *s* is said to be b-metric space.

### *Example 1*

(Boriceanu 2009c) The $l_{p}$ space, $0<p<1, l_{p}=\{(x_{n})\in {{\mathbb {R}}}:\sum |x_{n}|^{p}<\infty \}$ and function is defined as $d:l_{p}\times l_{p}\rightarrow {{\mathbb {R}}}$ by

$d(x,y)=\left(\sum |x_{n}-y_{n}|^{p}\right)^{\frac{1}{p}}, x=(x_{n}), y=(y_{n})\in l_{p}$ then (*X*, *d*) is said to b-metric space with parameter $s=2^{\frac{1}{2}}$ provided that $d(x,z)\le 2^{\frac{1}{2}}[d(x,y)+d(y,z)]$.

### *Example 2*

The space $L_{p}$ with $0<p<1$ of all real functions $x(t), t\in [0,1]$ such that $\int \nolimits _{0}^{1}|x(t)|^{p}<\infty$, if $d(x,y)=[\int \nolimits _{0}^{1}|x(t)-y(t)|^{p}dt]^{\frac{1}{p}}$ for all *x*, *y*$\in L_{p}$, then *d* satisfy all the condition of b-metric on the $L_{p}$ space.

### **Definition 2**

Boriceanu (2009c) Let (*X*, *d*) be a b-metric space. Then a sequence $\{x_{n}\}$ is said be converge to $x\in X$ if for each $\epsilon >0$ there exists $i(\epsilon )\in N$, such that $d(x_{n},x)<\epsilon$ for all $n\ge i(\epsilon )$.

### **Definition 3**

Boriceanu (2009c) Let (*X*, *d*) be a b-metric space. Then a sequence $\{x_{n}\}$ is said be a Cauchy sequence if for each $\epsilon >0$ there exists $i(\epsilon )\in N$, such that $d(x_{n}, x_{m})<\epsilon$ for all $n,m\ge i(\epsilon )$.

### **Definition 4**

Gnana Bhaskar and Lakshmikantham (2006) An element $(x,y)\in X\times X$ is said to be a coupled fixed point of $T:X\times X\rightarrow X$ if $x=T(x,y)$ and $y=T(y,x)$.

### **Definition 5**

An element $(x,y)\in X\times X$ is said to be a coupled coincidence point of $S,T:X\times X\rightarrow X$ if $S(x,y)=T(x,y)$ and $S(y,x)=T(y,x)$.

### *Example 3*

Suppose $X={{\mathbb {R}}}$ and $S,T:X\times X\rightarrow X$ defined as $S(x,y)=x^{2}y^{2}$ and $T(x,y)=(9/4)(x+y)$ for all $x, y\in X$. Then (3,1), (1,3) and (0,0) are coupled coincidence points of *S*, *T*.

### **Definition 6**

Let $S,T:X\times X\rightarrow X$ a point $(x,y)\in X\times X$ is said to be common fixed point of *S*, *T* if

$$\begin{aligned} x=S(x,y)=T(x,y)\ \ \hbox {and}\ \ \ y=S(y,x)=T(y,x). \end{aligned}$$

## Main results

This section derives some fixed point results in the setup of b-metric spaces.

### **Theorem 1**

*Let* (*X* , *d*) *be a completeb*-*metric space with parameter*$s \ge 1$*and let the mapping*$S,T: X\times X \rightarrow X$*satisfy:*

1 $$\begin{aligned} d(S(x,y),T(u,v))& \le \alpha _{1} \frac{d(x,u)+d(y,v)}{2}\nonumber \\ & \quad+\alpha _{2}\frac{d(x,S(x,y))d(u,T(u,v))}{1+d(x,u)+d(y,v)}\nonumber \\&\quad +\alpha _{3}\frac{d(u,S(x,y))d(x,T(u,v))}{1+d(x,u)+d(y,v)}\nonumber \\&\quad +\alpha _{4}\frac{d(S(x,y),T(u,v))d(x,u)}{1+d(x,u)+d(y,v)}\nonumber \\&\quad+\alpha _{5}\frac{d(S(x,y),T(u,v))d(y,v)}{1+d(x,u)+d(y,v)}\nonumber \\&\quad+\alpha _{6}\frac{d(u,T(u,v))d(y,v)}{1+d(x,u)+d(y,v)}\nonumber \\&\quad+\alpha _{7}\frac{d(u,S(x,y)d(x,u))}{1+d(x,u)+d(y,v)}\nonumber \\&\quad+\alpha _{8}\frac{d(u,S(x,y))d(y,v)}{1+d(x,u)+d(y,v)} \end{aligned}$$

*For all*$x,y,u,v,\in X$*and*$\alpha _{1},\alpha _{2},\alpha _{3},\alpha _{4},\alpha _{5},\alpha _{6},\alpha _{7},\alpha _{8}\ge 0$*with*$s\alpha _{1}+\alpha _{2}+\alpha _{4}+\alpha _{5}+\alpha _{6}<1$*and*$\alpha _{1}+\alpha _{3}+\alpha _{4}+\alpha _{5}+\alpha _{7}+\alpha _{8}<1.$*ThenSandThave unique common coupled fixed pointinX*.□

### *Proof*

Take two arbitrary points $$x_0, y_0$$ in *X*, define $$x_{2k+1}= S(x_{2k},y_{2k}), y_{2k+1}=S(y_{2k},x_{2k})$$, $$x_{2k+2}= T(x_{2k+1},y_{2k+1})$$, $$y_{2k+2}=T(y_{2k+1}$$, $$x_{2k+1})$$ for $$k= 0,1,2,\ldots .$$

Consider

$$\begin{aligned} d(x_{2k+1},x_{2k+2})=d(S(x_{2k},y_{2k}),T(x_{2k+1},y_{2k+1})). \end{aligned}$$

Then by using condition (1) of Theorem 1, we have

$$\begin{aligned} d(x_{2k+1},x_{2k+2})& \le \alpha _{1}\frac{d(x_{2k},x_{2k+1})+d(y_{2k},y_{2k+1})}{2}\\ & \quad+\alpha _{2}\frac{d(x_{2k},S(x_{2k},y_{2k})) d(x_{2k+1},T(x_{2k+1},y_{2k+1}))}{1+d(x_{2k},x_{2k+1})+d(y_{2k},y_{2k+1})}\\ & \quad +\alpha _{3}\frac{d(x_{2k+1},S(x_{2k},y_{2k}))d(x_{2k},T(x_{2k+1},y_{2k+1}))}{1+d(x_{2k},x_{2k+1})+ d(y_{2k},y_{2k+1})} \\ & \quad +\alpha _{4}\frac{d(S(x_{2k},y_{2k}),T(x_{2k+1},y_{2k+1}))d(x_{2k},x_{2k+1})}{1+d(x_{2k},x_{2k+1})+d(y_{2k},y_{2k+1})} \\ & \quad + \alpha _{5}\frac{d(S(x_{2k},y_{2k}),T(x_{2k+1},y_{2k+1}))d(y_{2k},y_{2k+1})}{1+d(x_{2k},x_{2k+1})+d(y_{2k},y_{2k+1})}\\ & \quad+\alpha _{6}\frac{d(x_{2k+1},T(x_{2k+1},y_{2k+1}))d(y_{2k},y_{2k+1})}{1+d(x_{2k},x_{2k+1})+d(y_{2k},y_{2k+1})}\\ & \quad +\alpha _{7}\frac{d(x_{2k+1},S(x_{2k},y_{2k}))d(x_{2k},x_{2k+1})}{1+d(x_{2k},x_{2k+1})+d(y_{2k},y_{2k+1})}\\ & \quad +\alpha _{8}\frac{d(x_{2k+1},S(x_{2k},y_{2k}))d(y_{2k},y_{2k+1})}{1+d(x_{2k},x_{2k+1})+d(y_{2k},y_{2k+1})}\\ & = \alpha _{1}\frac{d(x_{2k},x_{2k+1})+d(y_{2k},y_{2k+1})}{2}\\ & \quad+\alpha _{2}\frac{d(x_{2k},x_{2k+1})d(x_{2k+1},x_{2k+2})}{1+d(x_{2k},x_{2k+1})+d(y_{2k},y_{2k+1})}\\ & \quad +\alpha _{3}\frac{d(x_{2k+1},x_{2k+1})d(x_{2k},x_{2k+2})}{1+d(x_{2k},x_{2k+1})+d(y_{2k},y_{2k+1})}\\& \quad+ \alpha _{4}\frac{d(x_{2k+1},x_{2k+2})d(x_{2k},x_{2k+1})}{1+d(x_{2k},x_{2k+1})+d(y_{2k},y_{2k+1})}\\ & \quad+\alpha _{5}\frac{d(x_{2k+1},x_{2k+2})d(y_{2k},y_{2k+1})}{1+d(x_{2k},x_{2k+1})+d(y_{2k},y_{2k+1})}\\ & \quad+ \alpha _{6}\frac{d(x_{2k+1},x_{2k+2})d(y_{2k},y_{2k+1})}{1+d(x_{2k},x_{2k+1})+d(y_{2k},y_{2k+1})}\\ & \quad+\alpha _{7}\frac{d(x_{2k+1},x_{2k+1})d(x_{2k},x_{2k+1})}{1+d(x_{2k},x_{2k+1})+d(y_{2k},y_{2k+1})}\\ & \quad+\alpha _{8}\frac{d(x_{2k+1},x_{2k+1})d(y_{2k},y_{2k+1})}{1+d(x_{2k},x_{2k+1})+d(y_{2k},y_{2k+1})} \\ & \le \alpha _{1}\frac{d(x_{2k},x_{2k+1})}{2}+\alpha _{1}\frac{d(y_{2k},y_{2k+1})}{2}\\ & \quad +\alpha _{2}d(x_{2k+1},x_{2k+2})+ \alpha _{4}d(x_{2k+1},x_{2k+2})\\ &\quad +\alpha _{5}d(x_{2k+1},x_{2k+2})+\alpha _{6}d(x_{2k+1},x_{2k+2}). \end{aligned}$$

which implies that

$$\begin{aligned} (1-(\alpha _{2}+\alpha _{4}+\alpha _{5}+\alpha _{6}))d(x_{2k+1},x_{2k+2})\le \alpha _{1}\frac{d(x_{2k},x_{2k+1})}{2}+ \alpha _{1}\frac{d(y_{2k},y_{2k+1})}{2} \end{aligned}$$

2 $$\begin{aligned} d(x_{2k+1},x_{2k+2})& \le \alpha _{1}\frac{d(x_{2k},x_{2k+1})}{2(1-(\alpha _{2}+\alpha _{4}+\alpha _{5}+\alpha _{6}))}\nonumber \\ &\quad +\alpha _{1}\frac{d(y_{2k},y_{2k+1})}{2(1-(\alpha _{2}+\alpha _{4}+\alpha _{5}+\alpha _{6}))}. \end{aligned}$$

Proceeding similarly one can prove that

3 $$\begin{aligned} d(y_{2k+1},y_{2k+2})\le \alpha _{1}\frac{d(y_{2k},y_{2k+1})}{2(1-(\alpha _{2}+\alpha _{4}+\alpha _{5}+\alpha _{6}))}+ \alpha _{1}\frac{d(x_{2k},x_{2k+1})}{2(1-(\alpha _{2}+\alpha _{4}+\alpha _{5}+\alpha _{6}))}. \end{aligned}$$

Adding, (2) and (3), we get

$$\begin{aligned} d(x_{2k+1},x_{2k+2})+d(y_{2k+1},y_{2k+2})& \le \frac{\alpha _{1}}{(1-(\alpha _{2}+\alpha _{4}+\alpha _{5}+\alpha _{6}))}\\ & \quad [d(x_{2k},x_{2k+1})+d(y_{2k},y_{2k+1})] \\ & = h[d(x_{2k},x_{2k+1})+d(y_{2k},y_{2k+1})].\end{aligned}$$

where

$$\begin{aligned} h=\frac{\alpha _{1}}{(1-(\alpha _{2}+\alpha _{4}+\alpha _{5}+\alpha _{6}))}<1. \end{aligned}$$

Also,

4 $$\begin{aligned} d(x_{2k+2},x_{2k+3}) & \le \alpha _{1}\frac{d(x_{2k+1},x_{2k+2})}{2(1-(\alpha _{2}+\alpha _{4}+\alpha _{5}+\alpha _{6}))}\nonumber \\&\quad+\alpha _{1}\frac{d(y_{2k+1},y_{2k+2})}{2(1-(\alpha _{2}+\alpha _{4}+\alpha _{5}+\alpha _{6}))} \end{aligned}$$

5 $$\begin{aligned} d(y_{2k+2},y_{2k+3})& \le \alpha _{1}\frac{d(y_{2k+1},y_{2k+2})}{2(1-(\alpha _{2}+\alpha _{4}+\alpha _{5}+\alpha _{6}))}\nonumber \\ &\quad +\alpha _{1}\frac{d(x_{2k+1},x_{2k+2})}{2(1-(\alpha _{2}+\alpha _{4}+\alpha _{5}+\alpha _{6}))} \end{aligned}$$

Adding, (4) and (5), we get

$$\begin{aligned} d(x_{2k+2},x_{2k+3})+d(y_{2k+2},y_{2k+3}) & \le \frac{\alpha _{1}}{(1-(\alpha _{2}+\alpha _{4}+\alpha _{5}+\alpha _{6}))} \\ &\quad {[d(x_{2k+1},x_{2k+2})+d(y_{2k+1},y_{2k+2})]} \\ & = h[d(x_{2k+1},x_{2k+2})+d(y_{2k+1},y_{2k+2})]\\ & \le h^{2}[d(x_{2k},x_{2k+1})+d(y_{2k},y_{2k+1})]. \end{aligned}$$

Continuing this way, we have

$$\begin{aligned} d(x_{n},x_{n+1})+d(y_{n},y_{n+1}) & \le h[d(x_{n-1},x_{n})+d(y_{n-1},y_{n})]\\& \le h^{2}[d(x_{n-2},x_{n-1})+d(y_{n-2},y_{n-1})]\\ & \le \cdots \le h^{n}[d(x_{0},x_{1})+d(y_{0},y_{1})] \end{aligned}$$

If $$d(x_{n},x_{n+1})+d(y_{n},y_{n+1})=\delta _{n}$$ Then $$\delta _{n}\le h\delta _{n-1}\le h^{2}\delta _{n-2}\le \cdots \le h^{n}\delta _{0}.$$

For m $$>$$ n,

$$\begin{aligned}{}[d(x_{n},x_{m})+d(y_{n},y_{m})] & \le s[d(x_{n},x_{n+1})+d(y_{n},y_{n+1})] \\ & \quad +s^{2}[d(x_{n+1},x_{n+2})+d(y_{n+1},y_{n+2})]+\cdots \\&\quad+s^{m-n}[d(x_{m-1},x_{m})+d(y_{m-1},y_{m})]\\ & \le h^{n}s\delta _{0}+s^{2}h^{n+1}\delta _{0}+\cdots +s^{m-n}h^{m-1}\delta _{0} \\ & < sh^{n}[1+sh+(sh)^{2}+\cdots ]\delta _{0}\\ & = \frac{sh^{n}}{1-sh}\longrightarrow 0\hbox { as }n\longrightarrow \infty . \end{aligned}$$

Shows that $\{x_{n}\}$ and $\{y_{n}\}$ are Cauchy sequences in *X*. As *X* is complete *b*-metric space, so there exists $x,y \in X$ such that $x_{n} \longrightarrow x$ and $y_{n} \longrightarrow y$ as n$\longrightarrow \infty$.

Now we will prove that $x=S(x,y)$ and $y=S(y,x)$. On contrary suppose that $x\ne S(x,y)$ and $y\ne S(x,y)$. Then $d(x,S(x,y))=l_{1}>0$ and $d(y,S(x,y))=l_{2}>0$.

Consider the following and using condition (1) of Theorem 1, we get

$$\begin{aligned} l_{1}& = d(x,S(x,y))\le s[d(x,x_{2k+2})+d(x_{2k+2},S(x,y))]\\ & = sd(x,x_{2k+2})+sd(T(x_{2k+1},y_{2k+1}),S(x,y))\\ &= sd(x,x_{2k+2})+sd(S(x,y),T(x_{2k+1},y_{2k+1}))\\ & \le sd(x,x_{2k+2})+s\alpha _{1}\frac{d(x,x_{2k+1})+d(y,y_{2k+1})}{2}\\ &\quad+s\alpha _{2}\frac{d(x,S(x,y)) d(x_{2k+1},T(x_{2k+1},y_{2k+1}))}{1+d(x,x_{2k+1})+d(y,y_{2k+1})}\\&\quad+s\alpha _{3}\frac{d(x_{2k+1},S(x,y))d(x,T(x_{2k+1},y_{2k+1}))}{1+d(x,x_{2k+1}) +d(y,y_{2k+1})}\\&\quad+s\alpha _{4}\frac{d(S(x,y),T(x_{2k+1},y_{2k+1}))d(x,x_{2k+1})}{1+d(x,x_{2k+1})+d(y,y_{2k+1})}\\&\quad+s\alpha _{5}\frac{d(S(x,y),T(x_{2k+1},y_{2k+1}))d(y,y_{2k+1})}{1+d(x,x_{2k+1})+d(y,y_{2k+1})}\\&\quad+s\alpha _{6}\frac{d(x_{2k+1},T(x_{2k+1},y_{2k+1}))d(y,y_{2k+1})}{1+d(x,x_{2k+1})+d(y,y_{2k+1})}\\&\quad+s\alpha _{7}\frac{d(x_{2k+1},S(x,y))d(x,x_{2k+1})}{1+d(x,x_{2k+1})+d(y,y_{2k+1})} \\&\quad+s\alpha _{8}\frac{d(x_{2k+1},S(x,y))d(y,y_{2k+1})}{1+d(x,x_{2k+1})+d(y,y_{2k+1})}\\&= sd(x,x_{2k+2})+s\alpha _{1}\frac{d(x,x_{2k+1})+d(y,y_{2k+1})}{2}\\&\quad+s\alpha _{2}\frac{d(x,S(x,y)) d(x_{2k+1},x_{2k+2})}{1+d(x,x_{2k+1})+d(y,y_{2k+1})}\\&\quad+s\alpha _{3}\frac{d(x_{2k+1},S(x,y))d(x,x_{2k+2})}{1+d(x,x_{2k+1})+ d(y,y_{2k+1})}\\&\quad+s\alpha _{4}\frac{d(S(x,y),x_{2k+2})d(x,x_{2k+1})}{1+d(x,x_{2k+1})+d(y,y_{2k+1})}\\&\quad+s\alpha _{5}\frac{d(S(x,y),x_{2k+2}))d(y,y_{2k+1})}{1+d(x,x_{2k+1})+d(y,y_{2k+1})}\\&\quad+s\alpha _{6}\frac{d(x_{2k+1},x_{2k+2})d(y,y_{2k+1})}{1+d(x,x_{2k+1})+d(y,y_{2k+1})}\\&\quad+s\alpha _{7}\frac{d(x_{2k+1},S(x,y))d(x,x_{2k+1})}{1+d(x,x_{2k+1})+d(y,y_{2k+1})}\\&\quad+s\alpha _{8}\frac{d(x_{2k+1},S(x,y))d(y,y_{2k+1})}{1+d(x,x_{2k+1})+d(y,y_{2k+1})}. \end{aligned}$$

Since $$\{x_{n}\}$$ and $$\{y_{n}\}$$ are convergent to *x* and *y*, therefore by taking limit as $$k\rightarrow \infty$$ we get $$l_{1}\le 0.$$ Which is contradiction, so $$d(x,S(x,y))=0$$$$\Rightarrow x=S(x,y).$$

Similarly we can prove that $y=S(y,x)$. Also we can prove that $x=T(x,y)$ and $y=T(y,x)$, Thus (*x*, *y*) is a common coupled fixed point of *S* and *T*.

**Uniqueness**

Let $(x^{*},y^{*})\in \hbox {X}\times \, X$ be second common coupled fixed point of *S* and *T*.

Then by using condition (1) of Theorem 1, we have

$$\begin{aligned} d(x,x^{*}) & = d (S(x,y),T(x^{*},y^{*}))\\ & \le \alpha _{1}\frac{d(x,x^{*})+d(y,y^{*})}{2}+\alpha _{2}\frac{d(x,S(x,y) d(x^{*},T(x^{*},y^{*}))}{1+d(x,x^{*})+d(y,y^{*})}\\ & \quad \alpha _{3}\frac{d(x^{*},S(x,y))d(x,T(x^{*},y^{*}))}{1+d(x,x^{*})+ d(y,y^{*})}+\alpha _{4}\frac{d(S(x,y),T(x^{*},y^{*}))d(x,x^{*})}{1+d(x,x^{*})+d(y,y^{*})}\\ & \quad+\alpha _{5}\frac{d(S(x,y),T(x^{*},y^{*}))d(y,y^{*})}{1+d(x,x^{*})+d(y,y^{*})} +\alpha _{6}\frac{d(x^{*},T(x^{*},y^{*}))d(y,y^{*})}{1+d(x,x^{*})+d(y,y^{*})}\\ & \quad +\alpha _{7}\frac{d(x^{*},S(x,y))d(x,x^{*})}{1+d(x,x^{*})+d(y,y^{*})} +\alpha _{8}\frac{d(x^{*},S(x,y))d(y,y^{*})}{1+d(x,x^{*})+d(y,y^{*})} \\ & = \alpha _{1}\frac{d(x,x^{*})+d(y,y^{*})}{2}+\alpha _{2}\frac{d(x,x) d(x^{*},x^{*})}{1+d(x,x^{*})+d(y,y^{*})} \\ & \quad\alpha _{3}\frac{d(x^{*},x)d(x,x^{*})}{1+d(x,x^{*})+ d(y,y^{*})}+\alpha _{4}\frac{d(x,x^{*})d(x,x^{*})}{1+d(x,x^{*})+d(y,y^{*})}\\ & \quad+\alpha _{5}\frac{d(x,x^{*})d(y,y^{*})}{1+d(x,x^{*})+d(y,y^{*})} +\alpha _{6}\frac{d(x^{*},x^{*})d(y,y^{*})}{1+d(x,x^{*})+d(y,y^{*})} \\ & \quad+\alpha _{7}\frac{d(x^{*},x)d(x,x^{*})}{1+d(x,x^{*})+d(y,y^{*})} +\alpha _{8}\frac{d(x^{*},x)d(y,y^{*})}{1+d(x,x^{*})+d(y,y^{*})}\\ & \le \alpha _{1}\frac{d(x,x^{*})}{2}+\alpha _{1}\frac{d(y,y^{*})}{2}+\alpha _{3}d(x,x^{*})+\alpha _{4}d(x,x^{*})\\&\quad+\alpha _{5}d(x,x^{*})+\alpha _{7}d(x,x^{*})+\alpha _{8}d(x,x^{*}). \end{aligned}$$

Thus

$$\begin{aligned}&\left( 1-\frac{\alpha _{1}}{2}-\alpha _{3}-\alpha _{4}-\alpha _{5}-\alpha _{7}- \alpha _{8}\right) d(x,x^{*})\le \alpha _{1}\frac{d(y,y^{*})}{2}\\&\frac{(2-\alpha _{1}-2\alpha _{3}-2\alpha _{4} -2\alpha _{5}-2\alpha _{7}-2\alpha _{8})}{2} d(x,x^{*})\le \alpha _{1}\frac{d(y,y^{*})}{2} \end{aligned}$$

6 $$\begin{aligned} d(x,x^{*})\le \frac{\alpha _{1}}{(2-\alpha _{1}-2\alpha _{3}-2\alpha _{4}- 2\alpha _{5}-2\alpha _{7}-2\alpha _{8})}d(y,y^{*}). \end{aligned}$$

Similarly,

7 $$\begin{aligned} d(y,y^{*})\le \frac{\alpha _{1}}{(2-\alpha _{1}-2\alpha _{3}-2 \alpha _{4}-2\alpha _{5}-2\alpha _{7}-2\alpha _{8})}d(x,x^{*}). \end{aligned}$$

Adding, (6) and (7), we get

$$\begin{aligned} d(x,x^{*})+d(y,y^{*}) & \le \frac{\alpha _{1}}{(2-\alpha _{1}-2 \alpha _{3}-2\alpha _{4}-2\alpha _{5}-2\alpha _{7}-2\alpha _{8})} [d(y,y^{*})+d(x,x^{*})] \\ & \quad \left[ 1-\frac{\alpha _{1}}{(2-\alpha _{1}-2\alpha _{3}-2\alpha _{4}-2\alpha _{5}-2\alpha _{7}-2\alpha _{8})}\right] [d(y,y^{*})+d(x,x^{*})]\le 0\\ &\quad \frac{2(1-\alpha _{1}-\alpha _{3}-\alpha _{4}-\alpha _{5}-\alpha _{7}-\alpha _{8})}{2-\alpha _{1}-2\alpha _{3}-2\alpha _{4}- 2\alpha _{5}-2\alpha _{7}-2\alpha _{8}}[d(x,x^{*})+d(y,y^{*})]\le 0. \end{aligned}$$

Since $$\alpha _{1}+\alpha _{3}+\alpha _{4}+\alpha _{5}+\alpha _{7}+\alpha _{8}<1.$$

Therefore,

$$\begin{aligned} \frac{2(1-\alpha _{1}-\alpha _{3}-\alpha _{4}-\alpha _{5}-\alpha _{7} -\alpha _{8})}{2-\alpha _{1}-2\alpha _{3}-2\alpha _{4}- 2\alpha _{5}-2\alpha _{7}-2\alpha _{8}}>0. \end{aligned}$$

Hence

$$\begin{aligned}{}[d(x,x^{*})+d(y,y^{*})]\le 0. \end{aligned}$$

Which implies that $x=x^{*}$ and $y=y^{*} \Rightarrow (x,y)=(x^{*},y^{*})$.

Thus, *S* and *T* have unique common coupled fixed point.

Theorem 1 yields the following corollary.

### **Corollary 1**

*Let* (*X* , *d*) *be a completeb-metric space with parameter s*$\ge$*1 and let the mapping*$T: X\times X \rightarrow X$*mapping satisfy:*

$$\begin{aligned} d(T(x,y),T(u,v)) & \le \alpha _{1} \frac{d(x,u)+d(y,v)}{2}+\alpha _{2}\frac{d(x,T(x,y))d(u,T(u,v))}{1+d(x,u)+d(y,v)}\\&\quad+\alpha _{3}\frac{d(u,T(x,y))d(x,T(u,v))}{1+d(x,u)+d(y,v)}\\&\quad+\alpha _{4}\frac{d(T(x,y),T(u,v))d(x,u)}{1+d(x,u)+d(y,v)}\\&\quad+\alpha _{5}\frac{d(T(x,y),T(u,v))d(y,v)}{1+d(x,u)+d(y,v)}\\&\quad+\alpha _{6}\frac{d(u,T(u,v))d(y,v)}{1+d(x,u)+d(y,v)}\\ & \quad+\alpha _{7}\frac{d(u,T(x,y)d(x,u))}{1+d(x,u)+d(y,v)}\\ &\quad +\alpha _{8}\frac{d(u,T(x,y))d(y,v)}{1+d(x,u)+d(y,v)} \end{aligned}$$

*for all*$x,y,u,v,\in X$*and*$\alpha _{1},\alpha _{2},\alpha _{3},\alpha _{4},\alpha _{5},\alpha _{6},\alpha _{7},\alpha _{8}\ge 0$*with*$s\alpha _{1}+\alpha _{2}+\alpha _{4}+\alpha _{5}+\alpha _{6}<1$*and*$\alpha _{1}+\alpha _{3}+\alpha _{4}+\alpha _{5}+\alpha _{7}+\alpha _{8}<1.$*ThenThas unique common coupled fixed point inX*.

### *Proof*

The proof follows from Theorem 1 by taking $S=T$.□

### **Theorem 2**

*Let (X*, *d) be a complete b metric space with parameter*$s\ge 1$*and let the mapping*$S,T:X\times X\longrightarrow X$*satisfy:*

8 $$\begin{aligned} d(S(x,y),T(u,v)) &\le \alpha \frac{(d(x,u))+d(y,v)}{2}\nonumber \\&\quad+\beta \frac{d(x,S(x,y))d(u,T(u,v))}{1+s[d(x,T(u,v))+d(u,S(x,y))+d(x,u))+d(y,v)]}. \end{aligned}$$

*For all*$x,y,u,v\,\in$*X and *$\alpha ,\beta$*are non-negative real numbers with*$s(\alpha +\beta )<1$. *Then S andT have unique common coupled fixed point.*

### *Proof*

Take two arbitrary points $x_0, y_0$ in *X*. Define $x_{2k+1}=S(x_{2k},y_{2k}), y_{2k+1}=S(y_{2k},x_{2k}), x_{2k+2}=T(x_{2k+1},y_{2k+1})$ and $y_{2k+2}=T(y_{2k+1},x_{2k+1})$ for $k=0,1,2,\ldots$.

Consider

$$\begin{aligned} d(x_{2k+1},x_{2k+2})=d(S(x_{2k},y_{2k}),T(x_{2k+1},y_{2k+1})). \end{aligned}$$

Then by using condition (8) of Theorem 2, we have

$$\begin{aligned} d(x_{2k+1},x_{2k+2}) & \le \alpha \frac{d(x_{2k},x_{2k+1})+d(y_{2k},y_{2k+1})}{2}\\&\quad+\beta \frac{d(x_{2k},S(x_{2k},y_{2k}))d(x_{2k+1},T(x_{2k+1},y_{2k+1}))}{1+s[d(x_{2k},T(x_{2k+1},y_{2k+1}))+d(x_{2k+1},S(x_{2k},y_{2k}))+d(x_{2k},x_{2k+1})+d(y_{2k},y_{2k+1})]}\\ &= \alpha \frac{d(x_{2k},x_{2k+1})+d(y_{2k},y_{2k+1})}{2}\\&\quad+\beta \frac{d(x_{2k},x_{2k+1})d(x_{2k+1},x_{2k+2})}{1+s[d(x_{2k},x_{2k+2})+d(x_{2k+1},x_{2k+1})+d(x_{2k},x_{2k+1})+d(y_{2k},y_{2k+1})]}\\ &= \alpha \frac{d(x_{2k},x_{2k+1})}{2}+\alpha \frac{d(y_{2k},y_{2k+1})}{2}\\&\quad+\beta \frac{d(x_{2k},x_{2k+1})d(x_{2k+1},x_{2k+2})}{1+s[d(x_{2k+1},x_{2k+2})+d(y_{2k},y_{2k+1})]}\\ &\le \alpha \frac{d(x_{2k},x_{2k+1})}{2}+\alpha \frac{d(y_{2k},y_{2k+1})}{2}+\beta d(x_{2k},x_{2k+1}) \end{aligned}$$

which implies that

9 $$\begin{aligned} d(x_{2k+1},x_{2k+2})\le \frac{\alpha +2\beta }{2}d(x_{2k},x_{2k+1}) +\frac{\alpha }{2}d(y_{2k},y_{2k+1}). \end{aligned}$$

Similarly we can prove

10 $$\begin{aligned} d(y_{2k+1},y_{2k+2})\le \frac{\alpha +2\beta }{2}d(y_{2k},y_{2k+1}) +\frac{\alpha }{2}d(x_{2k},x_{2k+1}). \end{aligned}$$

Adding (9) and (10), we get

$$\begin{aligned}{}[d(x_{2k+1},x_{2k+2})+d(y_{2k+1},y_{2k+2})] \le (\alpha +\beta )[d(x_{2k},x_{2k+1})+d(y_{2k},y_{2k+1})]. \end{aligned}$$

Also

$$\begin{aligned} d(x_{2k+2},x_{2k+3})& = d(T(x_{2k+1},y_{2k+1}),S(x_{2k+2},y_{2k+2}))\\ & = d(S(x_{2k+2},y_{2k+2}),T(x_{2k+1},y_{2k+1}))\\ & \le \alpha \frac{d(x_{2k+2},x_{2k+1})+d(y_{2k+2},y_{2k+1})}{2}\\ & \quad+ \beta \frac{d(x_{2k+2},S(x_{2k+2},y_{2k+2}))d(x_{2k+1},T(x_{2k+1},y_{2k+1}))}{1+s[d(x_{2k+1},T(x_{2k+1},y_{2k+1}))+d(x_{2k+2},S(x_{2k+2},y_{2k+2}))+d(x_{2k+2},x_{2k+1})+d(y_{2k+2},y_{2k+1})]}\\ &= \alpha \frac{d(x_{2k+2},x_{2k+1})+d(y_{2k+2},y_{2k+1})}{2}\\&\quad+\beta \frac{d(x_{2k+2},x_{2k+3})d(x_{2k+1},x_{2k+2})}{1+s[d(x_{2k+1},x_{2k+2})+d(x_{2k+2},x_{2k+3})+d(x_{2k+2},x_{2k+1})+d(y_{2k+2},y_{2k+1})]}\\&\quad \Rightarrow d(x_{2k+2},x_{2k+3})\le \alpha \frac{d(x_{2k+2},x_{2k+2})}{2}+\alpha \frac{d(y_{2k+2},y_{2k+2})}{2}\\&\quad+ \beta \frac{d(x_{2k+2},x_{2k+3})d(x_{2k+1},x_{2k+2})}{1+s[d(x_{2k+1},x_{2k+3})+d(x_{2k+2},x_{2k+1})+d(y_{2k+2},y_{2k+1})]}\\ &= \alpha \frac{d(x_{2k+2},x_{2k+1})}{2}+\alpha \frac{d(y_{2k+2},y_{2k+1})}{2}+\beta \frac{d(x_{2k+2},x_{2k+3})d(x_{2k+1},x_{2k+2})}{1+s[d(x_{2k+2},x_{2k+3})+d(y_{2k+2},y_{2k+1})]}\\ \quad d(x_{2k+2},x_{2k+3}) & \le \alpha \frac{d(x_{2k+2},x_{2k+1})}{2}+\alpha \frac{d(y_{2k+2},y_{2k+1})}{2}+\beta d(x_{2k+1},x_{2k+2}) \end{aligned}$$

11 $$\begin{aligned} d(x_{2k+2},x_{2k+3})\le \frac{(\alpha +2\beta )}{2}{d(x_{2k+1},x_{2k+2})}+\alpha \frac{d(y_{2k+1},y_{2k+2})}{2} \end{aligned}$$

12 $$\begin{aligned} d(y_{2k+2},y_{2k+3})\le \frac{(\alpha +2\beta )}{2}{d(y_{2k+1},y_{2k+2})}+\alpha \frac{d(x_{2k+1},x_{2k+2})}{2}. \end{aligned}$$

Adding, (11) and (12), we get

$$\begin{aligned}{}[d(x_{2k+2},x_{2k+3})+d(y_{2k+2},y_{2k+3})] & \le (\alpha +\beta ) [d(x_{2k+1},x_{2k+2})+d(y_{2k+1},y_{2k+2})]\\ & \le (\alpha +\beta )^{2}[d(x_{2k},x_{2k+1})+d(y_{2k},y_{2k+1})] \end{aligned}$$

continuing the same process, we get

$$\begin{aligned} d(x_{n},x_{n+1})+d(y_{n},y_{n+1}) & \le (\alpha +\beta )[d(x_{n-1},x_{n})+d(y_{n-1},y_{n})]\\ & \le (\alpha +\beta )^{2}[d(x_{n-2},x_{n-1})+d(y_{n-2},y_{n-1})]\\ & \le \cdots \le (\alpha +\beta )^{n}[d(x_{0},x_{1})+d(y_{0},y_{1})] \end{aligned}$$

where $$h=\alpha +\beta <1$$.

Now if $d(x_{n},x_{n+1})+d(y_{n},y_{n+1})=\delta _{n}$. Then $\delta _{n}\le h\delta _{n-1}\le \cdots \le h^{n}\delta _{0}$

so for $m>n$, we have

$$\begin{aligned} d(x_{n},x_{m})+d(y_{n},y_{m}) & \le s[d(x_{n},x_{n+1})+d(y_{n},y_{n+1})]\\&\quad+\cdots +s^{m-n}[d(x_{m-1},x_{m})+d(y_{m-1},y_{m})]\\ & \le sh^{n}\delta _{0}+s^{2}h^{n+1}\delta _{0}+\cdots + s^{m-n}h^{m-1}\delta _{0}\\ &< sh^{n}[1+(sh)+(sh)^{2}+\cdots ]\delta _{0}\\& = \frac{sh^{n}}{1-sh}\delta _{0}\longrightarrow 0\hbox { as } n\longrightarrow \infty . \end{aligned}$$

Therefore, $\{x_{n}$} and $\{y_{n}\}$ are Cauchy sequences in *X*. Since *X* is complete *b*-metric space, there exists $x,y \in$*X* such that $x_{n}\longrightarrow x$ and $y_{n}\longrightarrow \, y$ as n$\longrightarrow \infty$.

Now we will show that $x=S(x,y)$ and $y=S(y,x)$. Suppose on contrary that $x\ne S(x,y)$ and $y\ne S(x,y)$, so that $d(x,s(x,y))=l_{1}>0$ and $d(y,s(x,y))=l_{2}>0$ consider the following and using condition (8) of Theorem 2, we get

$$\begin{aligned} l_{1} & = d(x,s(x,y))\le s[d(x,x_{2k+2})+d(x_{2k+2},S(x,y))]\\ & = sd(x,x_{2k+2})+sd(S(x,y),x_{2k+2})\\ & = sd(x,x_{2k+2})+sd(S(x,y),T(x_{2k+1},y_{2k+1}))\\ & \le sd(x,x_{2k+2})+s\alpha \frac{d(x,x_{2k+1})+d(y,y_{2k+1})}{2}\\ & \quad +s\beta \frac{d(x,S(x,y))d(x_{2k+1},T(x_{2k+1},y_{2k+1}))}{1+s[d(x,T(x_{2k+1},y_{2k+1}))+d(u,S(x,y)+d(x,x_{2k+1})+d(y,y_{2k+1})]}\\ & =sd(x,x_{2k+2})+s\alpha \frac{d(x,x_{2k+1})+d(y,y_{2k+1})}{2}\\&\quad+ s\beta \frac{d(x,S(x,y))d(x_{2k+1},x_{2k+2})}{1+s[d(x,x_{2k+2})+d(u,S(x,y))+d(x,x_{2k+1})+d(y,y_{2k+1})]}. \end{aligned}$$

Taking limit $k\rightarrow \infty$ we get $l_{1}\le 0$.

Therefore $d(x,S(x,y))=0$. Which implies that $x=S(x,y)$

Similarly we can prove that $y=S(y,x), x=T(x,y)$ and $y=T(y,x)$.

Hence (*x*, *y*) is a common coupled fixed point of *S* and *T*.□

**Uniqueness**

Let $(x^{*},y^{*})\in$ X$\times$ X be another common coupled fixed point of S and T.

Using condition (8) of Theorem 2 here, we get

$$\begin{aligned} d(x,x^{*}) & = d(S(x,y),T(x^{*},x^{*}))\le \alpha \frac{d(x,x^{*})+d(y,y^{*})}{2}\\&\quad+ \beta \frac{d(x,S(x,y))d(x^{*},T(x^{*},y^{*}))}{1+s[d(x,T(x^{*},y^{*}))+d(x^{*},S(x,y))+d(x,x^{*})+d(y,y^{*})]}\\ &\le \alpha \frac{d(x,x^{*})+d(y,y^{*})}{2}+\beta \frac{d(x,x)d(x^{*},x^{*})}{1+s[d(x,x^{*})+d(x^{*},x)+d(x,x^{*})+d(y,y^{*})]}\\ & = \alpha \frac{d(x,x^{*})}{2}+\alpha \frac{d(y,y^{*})}{2}+\beta \frac{d(x,x)d(x^{*},x^{*})}{1+s[3d(x,x^{*})+d(y,y^{*})]}. \end{aligned}$$

Therefore,

$$\begin{aligned} d(x,x^{*})& \le \alpha \frac{d(x,x^{*})}{2}+\alpha \frac{d(y,y^{*})}{2}\Rightarrow d(x,x^{*})\left[ 1-\frac{\alpha }{2}\right] \le \alpha \frac{d(y,y^{*})}{2}\\& \quad \Rightarrow d(x,x^{*})\left[ \frac{2-\alpha }{2}\right] \le \alpha \frac{d(y,y^{*})}{2} \end{aligned}$$

13 $$\begin{aligned} d(x,x^{*})\le \frac{\alpha }{2-\alpha }d(y,y^{*}). \end{aligned}$$

Similarly, we can prove that

14 $$\begin{aligned} d(y,y^{*})\le \frac{\alpha }{2-\alpha }d(x,x^{*}). \end{aligned}$$

Adding, (13) and (14), we get

$$\begin{aligned} d(x,x^{*})+d(y,y^{*})) & \le \frac{\alpha }{2-\alpha }[d(x,x^{*})+d(y,y^{*})]\\ &\quad \Rightarrow \left( 1-\frac{\alpha }{2-\alpha }\right) [d(x,x^{*})+d(y,y^{*})]\le 0 \end{aligned}$$

$d(x,x^{*})+d(y,y^{*})\le 0$, which implies that $x=x^{*}$ and $y=y^{*} \Rightarrow (x,y)=(x^{*},y^{*})$.

Hence, *S* and *T* have unique common coupled fixed point.

### **Corollary 2**

*Let (x*, *d) be a complete b metric space with parameter*$s\ge 1$*and let the mapping*$T:X\times X\Rightarrow X$*satisfy:*

$$\begin{aligned} d(T(x,y),T(u,v)) & \le \alpha \frac{(d(x,u))+d(y,v)}{2}\\& \quad+\beta \frac{d(x,T(x,y))d(u,T(u,v))}{1+s[d(x,T(u,v)) +d(u,T(x,y))+d(x,u)+d(y,v)]} \end{aligned}$$

*For all x,y,u,v*$\in$*X and*$\alpha ,\beta$*are non-negative real numbers with*$s(\alpha +\beta )<1$. *Then T has a unique common coupled fixed point.*

*Remarks*

- If $\alpha _{i}=0$ for $i=4,5,6,7,8$ in Theorem 1, then we get the result of Malhotra and Bansal (2015).
- If we take $S=T$ and $\alpha _{i}=0$ for $i=4,5,6,7,8$ in Theorem 1, then we get the corollary of Malhotra and Bansal (2015).

### *Example 4*

Suppose $X=[0,1]$. Defined the function $d:X\times X\rightarrow {{\mathbb {R}}}$ by $d(x,y)=\frac{2}{3}(x-y)^{2}\,\forall x,y\in X$. Clearly (*X*, *d*) is b-metric space with parameter $s=2$.

If we define $S,T:X\times X\rightarrow X$ by $S(x,y)=\frac{x+y}{2},T(x,y)=\frac{x+y}{3}$ for each $x, y \in X$. Then it can be proved simply that the maps *S* and *T* satisfy the conditions of Theorem 1 with $\alpha _{1}=\frac{1}{12},\alpha _{2}=\frac{1}{15}, \alpha _{3}=\frac{1}{6},\alpha _{4}=\frac{1}{9},\alpha _{5}=\frac{2}{15}, \alpha _{6}=\frac{1}{18},\alpha _{7}=\frac{5}{24},\alpha _{8}=\frac{5}{36}$. Hence (0,0) is a unique common coupled fixed point of *S* and *T*.

## Conclusion

The derived results generalize and extend some results of Malhotra and Bansal (2015) in the setting of b-metric spaces.
